# Behaviour and animal welfare indicators of broiler chickens housed in an enriched environment

**DOI:** 10.1371/journal.pone.0256963

**Published:** 2021-09-27

**Authors:** Marconi Italo Lourenço da Silva, Ibiara Correia de Lima Almeida Paz, Gustavo Henrique Coelho Chaves, Ianê Correia de Lima Almeida, Caio Cesar dos Ouros, Silvia Regina Lucas de Souza, Elisane Lenita Milbradt, Fabiana Ribeiro Caldara, Ana Júlia Garcia Satin, Gabriel Augusto da Costa, Andreia Soares Gonçalves Glavina

**Affiliations:** 1 Department of Animal Production and Preventive Veterinary Medicine, School of Veterinary Medicine and Animal Science (FMVZ), São Paulo State University “Júlio de Mesquita Filho” (UNESP), Botucatu, São Paulo, Brazil; 2 Department of Rural Engineering, School of Agronomic Science (FCA), São Paulo State University “Júlio de Mesquita Filho” (UNESP), Botucatu, São Paulo, Brazil; 3 Department of Animal Production, College of Agrarian Sciences, Federal University of Grande Dourados, Dourados, Mato Grosso do Sul, Brazil; Tokat Gaziosmanpasa Universitesi, TURKEY

## Abstract

The present study aimed to assess the influence of adding straw bales, step platforms, and laser projectors as environmental enrichment resources on the behaviour and welfare indicators of broiler chickens (*Gallus gallus domesticus*). A sample of 4,000 day-old male Cobb® 500 birds was used. The experimental treatments consisted of No Environmental Enrichment (NEE)—similar to a conventional environment; and Environmental Enrichment (EE)—environment enriched with straw bales, step platforms, and laser projectors, with four replicates per treatment of 500 animals. Behavioural characteristics (ethological observation through cameras, grab test, and modified touch test) and animal welfare indicators (pododermatitis and dorsal cranial myopathy) were assessed. The birds submitted to the EE treatment exhibited greater exploratory activity and expression of behaviours associated with comfort and welfare, whereas those in the NEE group were less active. Locomotion and play fighting behaviour decreased and behaviours associated with comfort increased as age advanced. The frequencies of interaction with laser spots and birds lying around straw bales were the highest in the 1^st^ week (P<0.01). The behaviours of pecking at straw bales (P<0.0004), using the step platforms (P = 0.0001) and being on top of straw bales (P<0.0002) gradually increased. The chickens accessed the feeding troughs the most in the period of 0800 hours (P<0.0001) and expressed the highest frequencies of behaviours associated with comfort in the 1400 hours and 1700 hours periods. The birds in the EE group were calmer in face of human presence and touch and scored higher in animal welfare indicators. Adding straw bales, step platforms, and laser projectors increased locomotion, reduced expression of fear, and improved animal welfare indicators of broiler chickens.

## Introduction

Environmental enrichment is a management strategy employed by researchers and the industry to increase the complexity of poultry barns and, consequently, increase the expression of natural behaviours by animals, thus improving their welfare [1]. According to Tahamtani et al. [2], adding resources as environmental enrichment breaks the monotony of the environment by making it more attractive and allowing animals to express their natural behaviour, in addition to aiding in reducing stress as the practice reduces fear and contribute to the development of cognitive functions such as learning and memory.

Behavioural expressions, i.e., fearful, panting, spatial distribution, dust bathing, scratching, pecking, and cannibalism, are used as indicators of animal welfare. Such behaviours reflect the emotional status of individuals [3].

Another major welfare indicator is animal locomotion. According to Garcia et al. [4], many behavioural patterns depend on locomotion, such as exploring the environment, seeking food, water, and shelter, and escaping predators. However, easy access to resources such as water, food, and shelter, associated with monotonous environments, high body weight, and high stocking density, interfere in walking ability and reduce the exploratory behaviour [4,5].

Concomitantly, rapid muscle growth and exacerbated development of the *Pectoralis major* muscle change the centre of gravity of broiler chickens, leading to skeletal-biomechanical imbalance [6,7]. As a behavioural response, birds tend to raise their wings to maintain balance, resulting in extended contraction of the anterior *Latissimus dorsi* muscle, which may cause issues such as dorsal cranial myopathy [8].

Furthermore, the rapid degradation of bedding in commercial farms using high stocking density and fast-growing lineages result in contact dermatitis in the plantar region of the feet, an inflammatory process called pododermatitis, which affects negatively on well-being [9,10]. Corrosive elements in the litter, such as excreta, are responsible for this condition. Pododermatitis may lead to secondary infections and cause partial carcass condemnation and significant economic losses as chicken feet command high prices in the foreign market [10,11].

Recent researches have reported positive effects of increased locomotion on the skeletal development of legs [12,13], which may improve animal welfare indicators. Locomotion can be increased through environmental enrichment using resources such as straw bales and platforms [14–17]. In addition, chickens tend to come near moving light spots and align their bodies in the same direction [18], which can be stimulated by the use of laser projectors. These light spots, provided by the laser projector, simulate insects found on the outside environment, favouring exploratory activity and, consequently, locomotion.

The use of straw bales is also associated with increased exploratory behaviour and improved welfare. Initially, birds use it as a protected resting area [19], which can reduce the stress of animals that feel threatened when performing resting and preening behaviours [20]. Then, the animals use it as an exploration area, increasing the frequency of the natural behaviours of pecking the straw bales. The dismantling of straw bales by birds leads to the addition of dry straw to the litter and increases foraging behaviour [19,21]. Straw bales are interesting as an enrichment resource, because they are practical and can be changed at each production cycle [21], however there is a need for good management of this resource to prevent the infestation by vectors into the barn, such as insects.

In addition to being practical, enrichment resources should be easy to sanitize, such as platforms that can be disinfected at the end of each production cycle [22]. The use of platforms as environmental enrichment is interesting because it presents fewer physical challenges to the animals [14]. In addition, they provide an elevated place that allows the birds to express the natural behaviour of surveillance against predators [23], being a favourable area for rest. According to Vasdal et al. [22], the use of platforms offered a variety of behaviours that may enhance the birds’ musculoskeletal strength and coordination, such as walking up and down, and jump off the platforms.

Some studies evaluating the use of straw bales and platforms have shown increased exploratory behaviour and locomotor activity [22,24,25], reduced incidence of pododermatitis [24], and reduced fear expression [26,27] when compared to animals housed in an environment without any environmental enrichment. However, other studies have not found these effects on exploratory behaviour [27,28] and incidence of pododermatitis [13,25,28]. This inconsistency may be related to the model and provision of these resources to animals, leading to different interactions and behaviour responses.

With that in mind, the present study aimed to assess the associate use of straw bales, step platforms, and laser projectors as environmental enrichment resources on the animal preference, behaviour, and welfare indicators of broiler chickens.

## Material and methods

The trial was carried out at the facilities of the School of Veterinary Medicine and Animal Sciences (FMVZ) of the São Paulo State University, Botucatu, SP, Botucatu (22° 49’ 07” S and 48° 24’ 40” W). The experimental protocol was approved by the Animal Use Ethics Committee of FMVZ (number 0092/2018 CEUA).

### Birds, facilities, and management

A sample of 4,000-day-old male Cobb® 500 birds from a commercial hatchery was used. The trial was carried out in a climate-controlled poultry barn (54 x 8 m) featuring a fully automated system with negative pressure ventilation using five exhaust fans and two cellulose evaporative panels. A 10 cm deep layer of new wood shavings was used as bedding. Feed was provided in automatic feeding (one feeder for fifty birds) troughs and nipple drinking (one nipple for ten birds) troughs. For this experiment, the barn was longitudinally delimited on two sides by a management corridor (54 x 2 m). On each side (54 x 3 m) there was a row of feeding troughs and a row of drinking troughs. The artificial lighting program 16L:8D during all experimental period was used. The diets, adapted from Rostagno [29], were prepared based on corn and soybean meal according to the nutritional requirements of the three rearing phases: initial (1–21 days, 24% CP and 3,000 kcal ME/kg), growth (22–35 days, 22.5% CP and 3,150 kcal ME/kg), and final (36–42 days, 19% CP and 3,250 kcal ME/kg). Both feed and water were provided *ad libitum*.

### Experimental design and treatments

**Fig 1:**
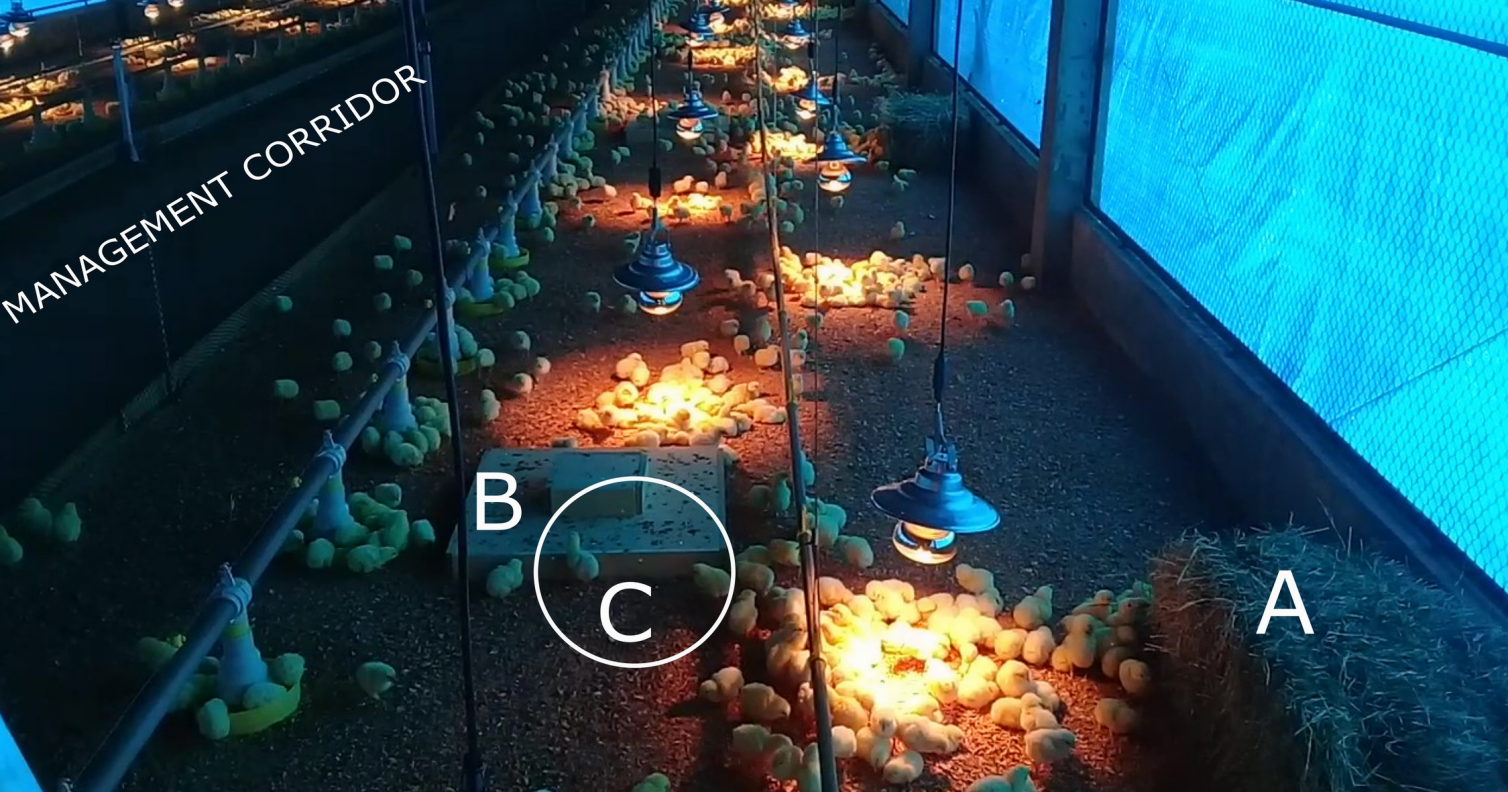
Birds housed in the environment containing environmental enrichment (EE). (A): Step platform, (B): Straw bale, (C): Laser spots.

The trial followed a completely randomized design with two treatments and four replicates each: No Environmental Enrichment (NEE): 2,000 birds housed in an environment similar to that found in commercial poultry´s house without any environmental enrichment; and Environmental Enrichment (EE): 2,000 birds housed in an environment similar to that found in commercial poultry´s house enriched with straw bales, step platforms, and laser projectors (Fig 1). Each replicate of treatments contained 500 birds, which were recorded. All animals were housed in the same poultry barn.

### Environmental enrichment resources

**Fig 2:**
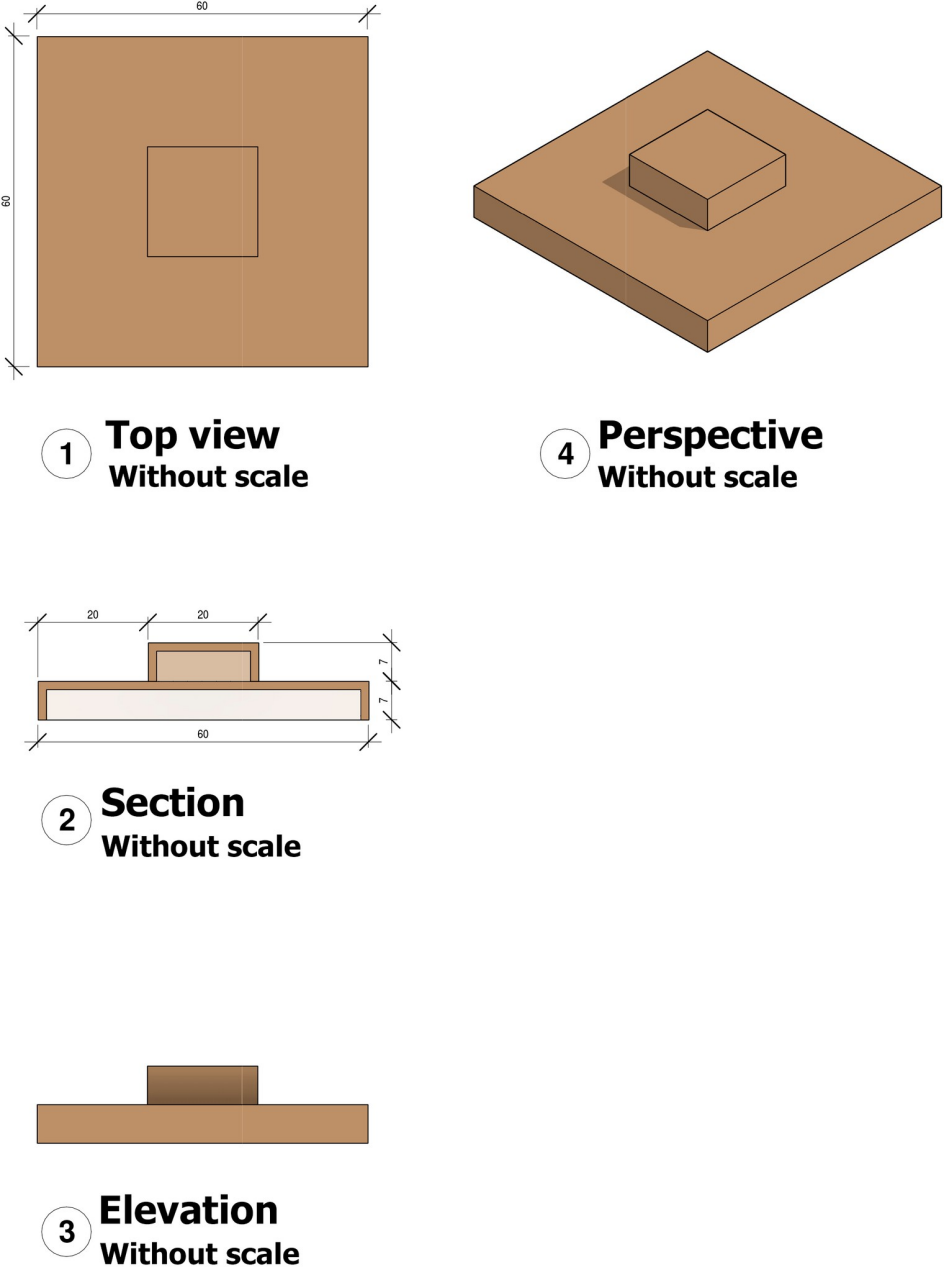
Step platform. (1): Top view with dimensions of the lower base, (2): Section with dimensions of the upper base and of the steps, (3): Elevation, (4) Perspective.

Straw bales (75 x 42 x 30 cm): one bale/500 birds. The straw bales were replaced by new ones at 35 days of the experimental period as they became worn and broken up due to intense use by the animals. Straw bales were placed between the drinkers’ row and the barn wall. Step platforms were made with MDF boards 60 x 60 x 7 cm in the lower base and 20 x 20 x 7 cm in the upper base (Fig 2). One platform was provided for every 500 birds. When the litter was turned, the platforms were scraped to remove excreta. Step platforms were placed between the drinkers’ row and the feeders’ row. Laser projectors–Mini Stage Lighting (13 x 9.2 x 5.2 cm) one projector for every 500 birds was used. The projectors emitted wavelengths of 532 nm (50 mW) and 650 nm (100 mW) the spots of light moved around an area of approximately 30 m^2^. A digital timer was used to turn on the projectors for 15 min in three periods (0800 hours, 1400 hours, and 1700 hours) for a total of 45 min of exposure per day. Light projectors were placed 1.5 m high in the same place where the cameras were. All resources were introduced on the first day and remained available until the end of the trial.

### Ethological observations

Eight high-resolution video cameras were placed along both longitudinal sides of each barn to form eight quadrants. Video was recorded over 24 h on days 6, 13, 20, 27, 34, and 41 of the experimental periods and the footage was analysed using the scan method. On the sixth day of recording, the camera images were observed for 15 min at 0800 hours, 1400 hours, and 1700 hours for a total of 45 min per day. The 15 min of each period were distributed into five observations comprising 1 min for each observation and 2 min of rest for a total of 720 observations. The times chosen for the analyses were the same as those when the animals were exposed to laser spots so that their behaviour could be assessed under the influence of all three environmental enrichment resources. The same observer performed all observations based on the ethogram adapted from Bergmann et al. [30] presented in Table 1. Each behaviour was individually assessed by the observer, and the frequency at which the behaviour was performed during the observation period was quantified.

**Table 1:** Experimental ethogram.

Behaviour	Operational definition
Lying/resting	Bird lies on the litter while the head rests on the ground or is erected; eyes may be open or shut
Locomotion	Bird moves at a fast or slow pace and occasionally flaps its wings
Grooming (self-grooming)	All behaviour patterns associated with cleaning and maintenance of its own body surface using the beak; the bird may stand or lie
Foraging while standing/scratching/pecking	Bird stands in upright position with both feet on the ground, uses both feet alternatively to paw at the ground, and/or lowers its head from time to time to peck at or move litter material in search of food
Eating	Bird with its head above the feeding trough or the surrounding area and actively taking in food
Drinking	Bird is actively taking in water by pecking at nipple drinkers or drinking out of the drip pan
Dust bathing	Combined preening and scratching behaviour (maintenance behaviour). Bird pecks and scratches at the litter material, then squats down onto the substrate and follows an organized sequence of behaviour patterns
Play fighting	After running against each other, the birds stop and face each other in a brief, non-harmful way. Behaviour is not persistently directed at any bird
*Behaviours associated with the provided environmental enrichment only*	
Lying around straw bales	Bird lies in immediate proximity to a straw bale
Pecking at straw bales	A single straw of the bale is pecked at and/or pulled out of it
Being on top of straw bales	Bird jumps, climbs, or flutters onto a straw bale and then lies or stands on top of it
Chasing and/or pecking at light spots	Bird chases and/or pecks at the light spots emitted by the laser projector
Using step platforms	Bird climbs onto a step platform and then lies down or stays on top of it

### Grab test

At 42 days, 5% of the birds of each treatment were randomly collected and individually placed inside a circle with 0.5 m radius, which was a new place for them. In order to minimize the influence of this change of environment, after 3 min the birds were grabbed, always by the same researcher. It was a blind test, and the researcher did not know which treatment the birds came from. The behaviours of the animals whilst being caught were scored 1 to 4 as adapted by Almeida Paz et al. [31], namely, 1: animal did not move; 2: animal walked away but did not vocalize or run; 3: animal walked away and vocalized; 4: animal ran/jumped and vocalized to try to avoid being caught.

### Modified touch test

The modified touch test was performed 1 h after the grab test. The researcher entered the experimental barn and waited for 2 min so that the birds could move around while in the presence of the person. After this time, the number of birds that could be reached by the immobile researcher were counted. The researcher did not make contact with the birds [31]. Twelve tests were performed distributed across four quadrants of each treatment.

### Pododermatitis

At 42 days, 5% of the birds of each treatment were randomly selected for assessment of feet condition. The measurements consisted in analysis of injuries to the footpad of the birds following the methodology described by Almeida Paz et. al. [10]. It was a blind test, and the researcher did not know which treatment the birds came from. Footpad integrity scores were 0: fully intact footpad; 1: initial lesion up to 5 mm in diameter, intermediate discomfort; and 2: extensive lesion with over 5 mm in diameter, imminent animal discomfort and reduced welfare.

### Dorsal cranial myopathy

At 43 days, 5% of the birds of each treatment were randomly selected and submitted to an 8 h fast. The birds were weighed and then stunned using a Fluxo UFX 7 electric stunner. The chickens were then exsanguinated via a cut to the carotid artery and jugular vein. After slaughter, dorsal cranial myopathy was macroscopically assessed in all animals. To that end, the *anterior latissimus dorsi* muscle was evaluated for uni- or bilateral integrity and scored as 0: intact muscle, with no apparent macroscopic lesions; 1: uni- or bilaterally affected muscle, with superficial haemorrhage, paleness, and gelatinous surroundings; and 2: muscle with uni- or bilateral altered colour exhibiting necrosis and increased volume. It was a blind test, and the researcher did not know which treatment the birds came from.

### Statistical analysis

The data were analysed using the statistical software SAS 9.2 (SAS Inst. Inc., Cary, NC, USA). The variance homogeneities were assessed by Levene’s test and data normality was verified by Shapiro-Wilk test. Behaviour data were subjected by ANOVA using MIXED procedure of SAS, followed by F-test or by a Tukey’s multiple comparison test and assigned significance when (P<0.05). The command REPEATED was applied, with the “compound symmetry covariance” structure being used for assessment of the effects on the measurements repeated in time (days). The interactions between the age x treatments and hour x treatments were included in the model, in addition to the effects of treatment, age and of hour alone.

Non-parametric statistics was applied to the data that did not meet the assumptions of the statistical model (normality and homogenicity) using Chi-squared (P<0.05) test or Fisher’s exact test (P<0.05) according to data behaviour.

## Results and discussion

### Behaviour

The birds housed in the enriched environment with straw bales, step platforms, and laser spots (EE) were, overall, more active than those housed in the conventional environment (NEE) as indicated by the greater number of animals in locomotion (P = 0.022) and smaller number of animals lying and resting (P = 0.005) (Table 2). The findings of this study corroborate other authors, who reported greater locomotion among birds when straw bales, platforms, and perches were provided, which shows those materials foster exploratory behaviour [17,22,24,25].

**Table 2:** Frequency (%) of each behaviour observed for broiler chickens as effects of the treatments.

Behaviour unit	Treatments		SE	ANOVA
	NEE	EE		
Lying/resting	72.28 a	60.10 b	2.87	*F*1,6 = 17.83, *P* = 0.005
Locomotion	3.41 b	5.03 a	0.51	*F*1,6 = 9.47, *P* = 0.022
Grooming (self-grooming)	3.86	3.77	0.23	*F*1,6 = 0.12, *P* = 0.737
Foraging while standing/scratching/pecking	4.76	4.64	0.34	*F*1,6 = 0.30, *P* = 0.601
Eating	12.07	11.37	1.41	*F*1,6 = 0.22, P = 0.652
Drinking	3.13	3.68	0.41	*F*1,6 = 1.27, *P* = 0.302
Dust bathing	0.12 b	0.27 a	0.06	*F*1,6 = 4.58, *P* = 0.046
Play fighting	0.37 a	0.21 b	0.05	*F*1,6 = 8.90, *P* = 0.024
*Behaviours associated with the provided environmental enrichment only*				
Lying around straw bales	-	5.06	0.27	-
Pecking at straw bales	-	1.69	0.08	-
Being on top of straw bales	-	0.44	0.13	-
Chasing and/or pecking at light spots	-	0.75	0.07	-
Using step platforms	-	3.00	0.45	-

Frequencies followed by “a, b” in the rows differ according to F-test (P<0.05). NEE: No Environmental Enrichment; EE: Environmental Enrichment.

The birds in the NEE treatment exhibited a higher frequency of the play fighting behaviour (P = 0.024), which may be associated with a monotonous environment since broiler chickens are not considered aggressive and are too young to have a complete dominance hierarchy [32]. The birds in the EE treatment exhibited a higher frequency of dust bathing (P = 0.046), which is associated with comfort and well-being in chickens, a natural behaviour for the species that is important for feather maintenance [33].

**Fig 3:**
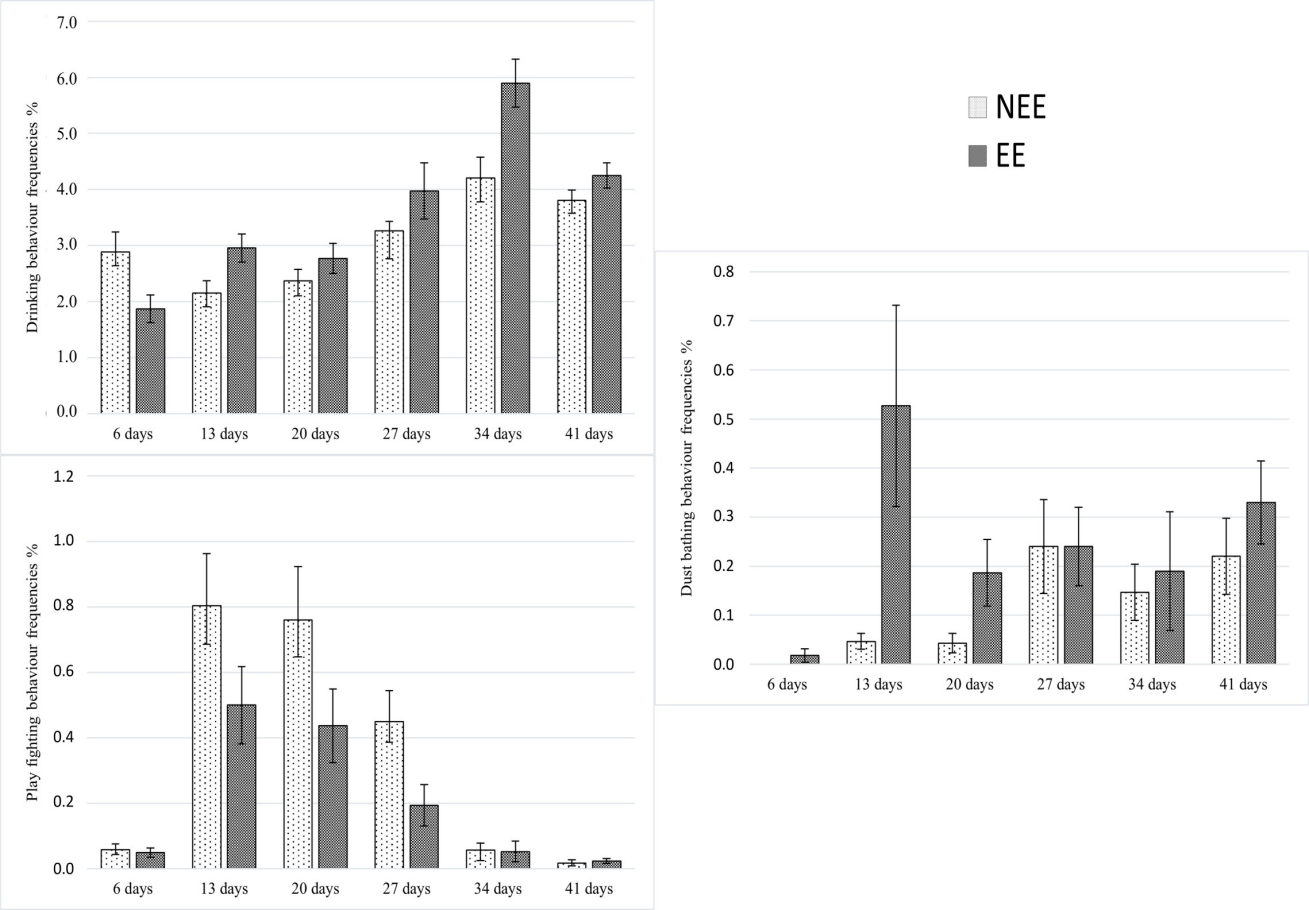
The effect of age on the frequencies (%) of behaviours performed in the NEE and EE treatments. (A): Drinking, (B): Dust bathing, (C): Play fighting. See more information in the supplementary material.

The effects of age in the frequency of each behaviour observed are presented in Table 3. There were no significant interactions between treatment and period in the behaviours Lying/resting (P = 0.557), Locomotion (P = 0.052), Grooming (P = 0.479), Foraging while standing/scratching/pecking (P = 0.211), and Eating (0.094). There were significant interactions between treatment and period in the behaviours drinking (*F*5,28 = 7.14, P = 0.0002); dust bathing (*F*5,28 = 2.89, *P =* 0.032), and play fighting (*F*5,28 = 2.92, *P* = 0.030) (Fig 3).

**Table 3:** Frequency (%) of each behaviour observed for broiler chickens as effects of age.

Behaviour unit	Age (days)						SE	ANOVA	Treat X age
	6	13	20	27	34	41			
Lying/resting	63.39	64.04	63.86	63.57	61.20	68.76	1.71	*F*5,28 = 6.98, *P* = 0.090	0.557
Locomotion	4.76 a	5.12 a	4.91 a	4.17 ab	3.18 bc	2.45 c	0.33	*F*5,28 = 21.12, *P*<0.0001	0.052
Grooming (self-grooming)	1.75 c	3.52 b	3.41 b	3.57 b	4.69 a	4.86 a	0.19	*F*5,28 = 38.78, *P*<0.0001	0.479
Foraging while standing/scratching/pecking	2.25 c	4.12 b	4.87 ab	4.72 ab	5.37 a	4.93 ab	0.24	*F*5,28 = 22.60, *P*<0.0001	0.211
Eating	10.24 ab	12.15 a	12.23 a	11.01 ab	13.77 a	8.6 b	0.80	*F*5,28 = 15.99, *P*<0.0001	0.094
Drinking	2.19 c	2.48 c	2.51 c	3.51 b	4.91 a	3.96 b	0.25	*F*5,28 = 37.38, *P*<0.0001	0.0002
Dust bathing	0.01 b	0.29 a	0.11 ab	0.23 ab	0.17 ab	0.27 a	0.06	*F*5,28 = 3.61, *P* = 0.012	0.032
Play fighting	0.05 c	0.62 a	0.58 a	0.31 b	0.05 c	0.02 c	0.05	*F*5,28 = 36.10, *P*<0.0001	0.030

Frequencies followed by “a, b, c” in the rows differ according to Tukey’s test (P<0.05).

As their age advanced, the animals became less active, reducing the percentage of the locomotion behaviour, with effects starting at 34 days (P<0.0001). It is possible that, as they became heavier, the birds reduced locomotion. Modern chicken lineages have a too-fast growth, consequently, the skeletal and muscle development is not enough to support the heavy weight, which may have led to the gradual decrease in locomotion. According to Bailie et al. [15], the activity of chickens of fast-growing lineages is reduced in the final stages of production. The present results corroborate the findings by Bergmann et al. [30], who tested a fast-growing lineage in two different rearing system and observed reduced locomotion as age advanced. Other authors also found reduced locomotion in the growth and finishing phases of broilers when assessing straw bales, perches, and platforms [21,22,24,25].

**Fig 4:**
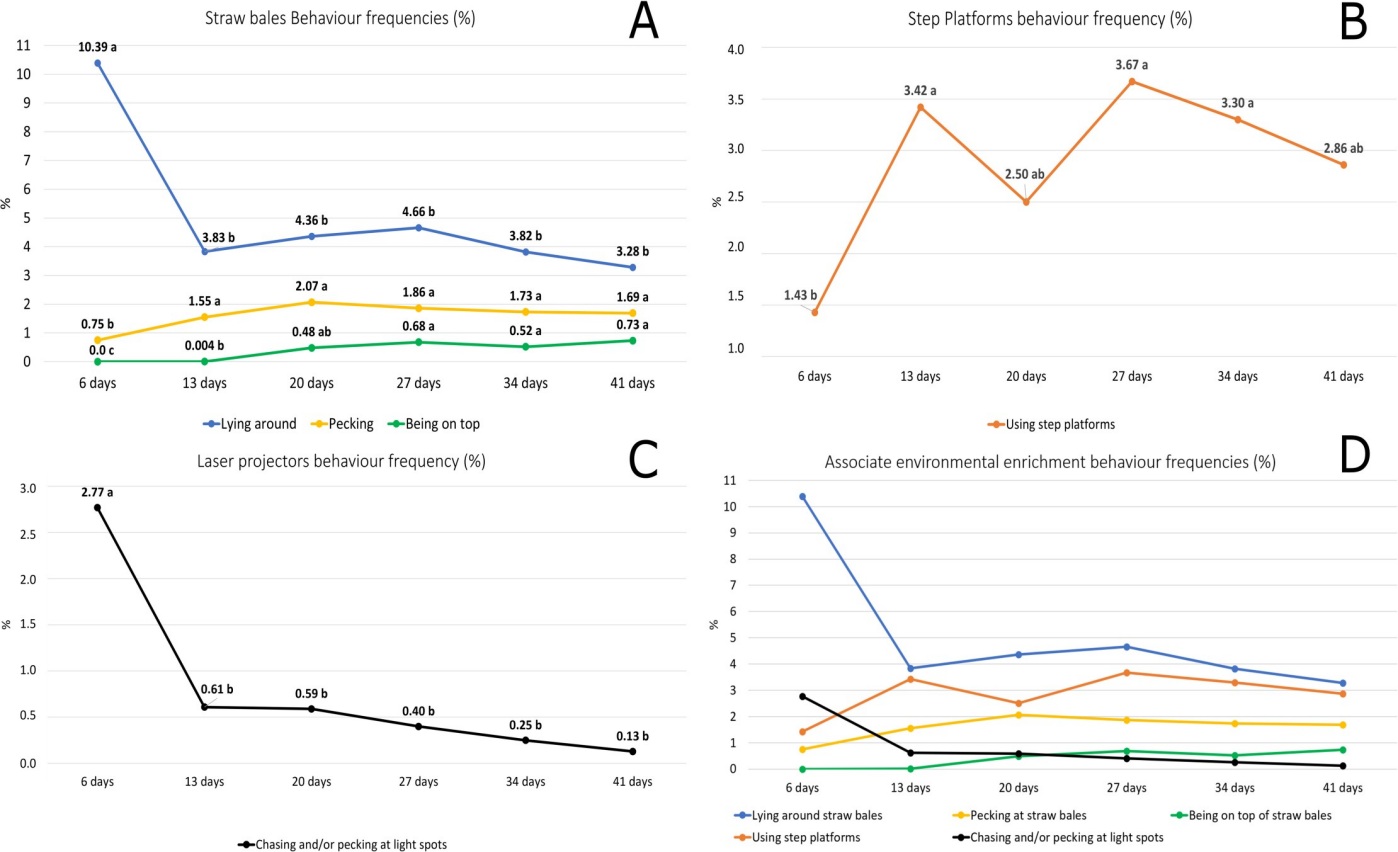
Frequencies (%) of behaviours associated with the provided environmental enrichment only. (A): Straw bales behaviour frequencies–Lying around (*F*5,14 = 17.15, *P*<0.0001), Pecking (*F*5,14 = 9.76, P = 0.0004), and Being on top (*F*5,14 = 11.24, P = 0.0002). (B): Step platforms behaviour frequency–Using step platforms (*F*5,28 = 12.03, *P*<0.0001). (C): Laser projectors behaviour frequency—Chasing and/or pecking at light spots (*F*5,14 = 13.51, *P*<0.0001). (D): Associate environmental enrichment behaviour frequencies.

The increase in animal inactivity over time resulted in a higher expression of other behaviours that do not necessarily involve locomotion, such as hygiene (self-grooming) (P<0.0001), foraging while standing/scratching/pecking (P<0.0001), dust bathing (P = 0.012), and pecking at straw bales (SE = 0.14, F5,14 = 9.76, P = 0.0004) (Fig 4). The expression of other behaviours decreased as age advanced and the animals grew inactive, such as play fighting (P<0.0001), chasing and/or pecking at light spots (SE = 0.25, F5,14 = 13.51, P<0.0001), and lying around straw bales (SE = 0.64, F5,14 = 17.15, P<0.0001). Hygiene (self-grooming) and foraging while standing/scratching/pecking are natural maintenance behaviours of the species. The increase in those behaviours, associated with the reduction in the number of birds lying around straw bales and the increase in the number of those pecking at straw bales may be the result of reduced expression of fear of the environment [34]. Under natural conditions, birds tend to seek a sheltered place for rest, which is also observed in modern chicken lineages [17]. Bergmann et al. [30] also found a lower frequency of chickens lying around straw bales as age advanced. As the frequency of that behaviour decreased, the expression of pecking at the straw bales increases, i.e., the bales ceased being objects for protection to become objects for exploration. The reduction in play fighting took place concomitantly to the reduction in activity as age advanced. This behaviour was possibly influenced by the birds in the NEE treatment, whose environment lacked exploratory stimuli. Thus, the animals in that treatment expressed that behaviour more often than the animals in EE treatment. Nonetheless, as their weight increased, such behaviour naturally decreased. Other authors have also found a lower expression of that behaviour as age increased [22,25].

The increase in the frequency of birds using steps platforms (SE = 0.48, F5,14 = 12.03, P = 0.0001) and being on top of straw bales (SE = 0.15, F5,14 = 11.24, P = 0.0002) may be the result of the increase in exploratory activity promoted by the association of environmental enrichment resources and a favourable resting area as age advanced. Birds started climbing onto straw bales in the second week of life. Initially, the bales were used as protection and shelter, with a larger number of animal groups lying and resting around them. Next, bales were used for exploration, with a higher frequency of birds pecking at them. Concomitantly, the bales were used as rest areas, with a higher frequency of animals on top of the objects. It is possible that the association between environmental enrichment resources may have mitigated the effects of the skeletal-biomechanical imbalance of birds by improving leg health with the increase of locomotion [6,12,14], this may have allowed the animals to access the top of the straw bales more often as age advanced. Straw bales, in an barn environment, seem to be a favourable resource for raised rest areas to fulfil the innate behaviour of the species of avoiding predators [23]. The present results corroborate the findings by Bergmann et al. [30], who reported a higher percentage of birds on top of straw bales as age advanced.

The frequency of the behaviour of chasing and/or pecking at the light spots was higher in the first week and significantly decreased in the second week of life. Possibly, the light spots lost their novelty and became less attractive to animals, in addition, this resource does not provide a reward such as the light spots being captured by the birds. On the other hand, the other objects such as straw bales and step platforms rewarded the animals, whether by pulling out straw from the bales or by the higher position on top of the platforms, which led to an increase in the frequency with which the birds used those resources as age advanced.

The step platforms were designed aiming at making the animals involuntarily exercise so as to strengthen their locomotive system and improve leg health. As the animals increased their exploratory activity, the use of platforms also increased, which shows this type of resource is well accepted. According to Kaukonen et al. [14], chickens prefer to use platforms over perches likely due to the greater physical challenges they face with the latter. Bailie et al. [35], when testing three types of perches, found the shape of a platform to be the most used. When evaluating perches, other authors have also reported greater use as age advances [35–38].

It is known that foraging while standing, scratching, pecking, and dust bathing are specific behaviours of the species and are associated with comfort and maintenance [21,33]. Those behaviours were most often observed at 1400 hours and 1700 hours. On the other hand, the frequency of the animals by the feeding troughs was the highest at 0800 hours (P<0.0001) (Table 4). That may be related to their circadian rhythm since, the barn lights were turned on at 0600 hours, it can have stimulated feed intake around the time of observation. The animals were, overall, more active at 0800 hours as indicated by the greater number of animals in locomotion (P = 0.006) and chasing and/or pecking at light spots (P = 0.038). Besides, other behaviours like lying and resting (P = 0.006) and lying around straw bales (P<0.0001) were performed more often at 1400 hours and 1700 hours. The results corroborate the findings by Jong and Gunnink [25], who reported greater expression of behaviours associated with comfort (pecking, scratching, and dust bathing) in the afternoon. There were no significant interactions between treatment and period in all behaviours evaluated (P>0.05).

**Table 4:** Frequency (%) of each behaviour observed for broiler chickens as effects of time of day.

Behaviour unit	Period			SE	ANOVA	Treat X period
	0800 hours	1400 hours	1700 hours			
Lying/resting	61.06 b	64.36 a	62.91 a	1.49	*F*2,12 = 7.92, *P* = 0.006	0.235
Locomotion	4.35 a	3.67 b	3.96 b	0.28	*F*2,12 = 6.45, *P* = 0.012	0.205
Grooming (self-grooming)	3.94	3.48	3.43	0.14	*F*2,12 = 5.31, *P* = 0.122	0.233
Foraging while standing/scratching/pecking	3.87 b	4.56 a	4.91 a	0.20	*F*2,12 = 11.67, *P* = 0.001	0.566
Eating	13.55 a	9.62 b	10.27 b	0.18	*F*2,12 = 49.91, *P*<0.0001	0.232
Drinking	3.20	3.25	3.21	0.23	*F*2,12 = 0.07, *P* = 0.935	0.211
Dust bathing	0.05 b	0.22 a	0.27 a	0.04	*F*2,12 = 9.01, *P* = 0.004	0.557
Play fighting	0.25	0.21	0.36	0.04	*F*2,12 = 5.84, *P* = 0.1069	0.289
*Behaviours associated with the provided environmental enrichment only*						
Lying around straw bales	3.70 b	5.43 a	5.16 a	0.57	*F*2,6 = 6.54, *P<*0.0001	-
Pecking at straw bales	1.65	1.60	1.57	0.11	*F*2,6 = 0.50, *P* = 0.631	-
Being on top of straw bales	0.48	0.39	0.37	0.14	*F*2,6 = 0.45, *P* = 0.655	-
Chasing and/or pecking at light spots	1.21 a	0.43 b	0.54 b	0.15	*F*2,6 = 5.95, *P* = 0.038	-
Using step platforms	2.68	2.79	3.04	0.45	*F*2,6 = 1.05, *P* = 0.405	-

Frequencies followed by “a, b” in the rows differ according to Tukey’s test (P<0.05).

The scores of the modified touch test and grab test are presented in Table 5. The birds housed in the enriched environment (EE) exhibited a higher frequency in the modified touch test compared to those in the conventional environment (NEE), meaning a higher number of chickens could be reached (P<0.0001). The animals in the EE treatment also exhibited a higher frequency of score 1 (animal did not move) in the grab test (P = 0.0027), which shows they were calmer and expressed less fear to human presence and touch, whereas those in the NEE treatment exhibited a higher frequency of score 3 (animal walked away and vocalized), showing they were more agitated. In practice, these findings may facilitate catching animals for slaughter in commercial poultry barns.

**Table 5:** Frequencies of the modified touch test and of scores in the grab test of broiler chickens housed in environments with and without enrichment at 42 days.

Modified touch test (%)	Treatments	
	NEE	EE
Birds that could be touched	2.27 b	5.87 a
	Chi-squared test: *x*^2^ 2 = 28.01, *P*<0.0001	
**Grab test (scores)**	**NEE**	**EE**
1	42.86 b	66.67 a
2	23.81	23.81
3	28.57 a	4.76 b
4	4.76	4.76
	Fisher’s exact test: *P* = 0.005	

Grab test: 1 –animal did not move; 2 –animal walked away but did not vocalize or run; 3 –animal walked away and vocalized; 4 –animal ran/jumped and vocalized to try to avoid being caught. Frequencies in the modified touch test and grab test followed by different letters in the rows differ. NEE: No Environmental Enrichment; EE: Environmental Enrichment.

Environmental enrichment elements are associated with reduced fear since animals reared with access to them are better able to deal with physiological and behavioural challenges [39], such as human presence [2]. Other studies have also observed lower expression of fear by broiler chickens when environmental complexity increased with the use of various enrichment resources such as perches, ropes, mirrors, and balls [40–43].

### Animal welfare indicators

By the end of 42 days of rearing, the birds housed in NEE treatment presented 3083 g of body weight, and the birds housed in EE treatment present 3089 g of body weight. The densities were calculated at 37.75 kg/m^2^ and 36.90 kg/m^2^ for the EE and NEE treatments, respectively. No differences in body weight or densities were found (P>0.05).

The frequencies of pododermatitis and dorsal cranial myopathy are presented in Table 6. One of the main causes of pododermatitis are corrosive factors in the litter, which worsen as the bedding is degraded over the production period [9]. Nevertheless, the birds in the EE group exhibited a higher frequency of score 0 (fully intact footpad), whereas those in the NEE group exhibited a higher frequency of score 2 (extensive lesion over 5 mm in diameter) (P = 0.0100). The presence of the step platforms and straw bales may have decreased the number of birds exposed to the degraded bedding since, over time, the number of animals using the platforms (P<0.0001) and climbing onto the bales (P<0.0001) increased (Table 2). The Straw bales were replaced by new ones at 35 days of the experimental period, and when the litter was turned, the platforms were scraped to remove excreta. Similar results were found by other authors when testing elevated platforms, perches and, straw bales as environmental enrichment [24,44,45]. However, other researchers found no effects on the frequency of pododermatitis when comparing animals reared in enriched and conventional environments [13,21,25,28,35,38,46,47], which may have been an influence of the design, type, or placement of the environmental enrichment resources in the aviaries.

**Table 6:** Frequencies (scores) of pododermatitis and dorsal cranial myopathy in broiler chickens housed in environments with and without enrichment.

**Pododermatitis**	**Treatments**	
	**NEE**	**EE**
0	34.78 b	55.27 a
1	30.43	39.47
2	34.79 a	5.26 b
	Chi-squared test: *x*^*2*^ 2 = 9.20, *P* = 0.0100	
**Cranial dorsal myopathy**	**NEE**	**EE**
0	24.70 b	34.12 a
1	44.71 b	54.94 a
2	30.59 a	10.94 b
	Chi-squared test: *x*^*2*^ 2 = 7.95, *P* = 0.0188	

Pododermatitis: 0 –fully intact footpad; 1 –initial lesion up to 5 mm in diameter; and 2 –extensive lesion over 5 mm in diameter. Dorsal cranial myopathy: 0 –intact muscle, with no apparent macroscopic lesions; 1 –uni- or bilaterally affected muscle, with superficial haemorrhage, paleness, and gelatinous surroundings; and 2 –muscle with uni- or bilateral altered colour exhibiting necrosis and increased volume. Frequencies followed by different letters in the rows differ. NEE: No Environmental Enrichment; EE: Environmental Enrichment.

Dorsal cranial myopathy—a lesion in the cranial portion of the back of broiler chickens, in the anterior *Latissimus dorsi* muscle, which is responsible for supporting the abduction of the humerus and wing [48]–is a growing cause of carcass condemnation in slaughterhouses in Brazil and the United States. In the present study, the animals in the EE treatment exhibited higher frequencies of scores 0 (intact muscle, with no apparent macroscopic lesions) and 1 (muscle with superficial haemorrhage, paleness, and gelatinous surroundings) than those in the NEE group, which exhibited a higher frequency of score 2 (muscle with uni- or bilateral altered colour exhibiting necrosis and increased volume) (P = 0.0188). It is likely that the greater level of activity of the animals in the EE treatment, associated with the use of step platforms, may have strengthened their locomotive system, thus preventing the skeletal-biomechanical imbalance caused by exacerbated growth of the *Pectoralis major* muscle in modern chicken lineages [6,7]. The higher frequency of calmer birds in the EE treatment may also be associated with the reduced incidence of dorsal cranial myopathy since chickens with greater expression of fear to human approximation and touch tend to escape while flapping their wings. According to Coates [49], a possible cause of this lesion is excessive wing flapping.

## Conclusion

The use of straw bales, step platforms, and laser projectors as environmental enrichment resources increases the locomotion of broiler chickens and the expression of natural behaviours of the species associated with comfort and welfare. Birds reared in enriched environments are calmer and express less fear. Straw bales, step platforms, and light projectors are useful in strengthening the locomotive system and in decreasing the incidence of locomotion issues such as pododermatitis and dorsal cranial myopathy.

## Supporting information

S1 Appendix The effect of age on the frequencies (%) of behaviours performed in the NEE and EE treatments (Supplementary material for Fig 3). (DOCX)
