# Supplementary material for: Behaviour and animal welfare indicators of broiler chickens housed in an enriched environment
Source: PLoS One. 2021 Sep 27;16(9):e0256963. doi: 10.1371/journal.pone.0256963 (PMC8476007; doi:10.1371/journal.pone.0256963)
Supplement: S1 Appendix — (DOCX) [file pone.0256963.s001.docx]

**S1 Appendix. Appendix 1. The effect of age on the frequencies (%) of behaviours performed in the NEE and EE treatments (Supplementary material for Fig 3)**

| **Age** | **Drinking** | | **Dust bathing** | | **Play fighting** | |
| --- | --- | --- | --- | --- | --- | --- |
|  | NEE | EE | NEE | EE | NEE | EE |
| 6 days | 2.88±0.36 a | 1.87±0.25 b | 0.00±0.00 | 0.02±0.01 | 0.06±0.02 | 0.05±0.01 |
| 13 days | 2.16±0.22 | 2.96±0.25 | 0.05±0.02 b | 0.53±0.20 a | 0.80±0.16 a | 0.50±0.12 b |
| 20 days | 2.37±0.20 | 2.77±0.27 | 0.04±0.02 | 0.19±0.07 | 0.76±0.16 a | 0.44±0.11 b |
| 27 days | 3.27±0.17 | 3.97±0.50 | 0.24±0.10 | 0.24±0.08 | 0.45±0.09 a | 0.19±0.06 b |
| 34 days | 4.21±0.37 b | 5.90±0.43 a | 0.15±0.06 | 0.19±0.12 | 0.06±0.02 | 0.05±0.03 |
| 41 days | 3.80±0.18 | 4.25±0.23 | 0.22±0.08 | 0.33±0.08 | 0.02±0.01 | 0.02±0.01 |
| **ANOVA** | *F*5,28=7.14, *P*=0.0002 | | *F*5,28=2.89, *P=*0.032 | | *F*5,28=2.92, *P*=0.030 | |

Frequencies followed by “a, b” in the rows of each behaviour differ according to Tukey’s test (P<0.05). NEE = No Environmental Enrichment. EE = Environmental Enrichment.
